# Heart over mind: metabolic control of white adipose tissue and liver

**DOI:** 10.15252/emmm.201404749

**Published:** 2014-12-03

**Authors:** Michinari Nakamura, Junichi Sadoshima

**Affiliations:** Department of Cell Biology and Molecular Medicine, Cardiovascular Research Institute, Rutgers-New Jersey Medical SchoolNewark, NJ, USAE-mail: sadoshju@njms.rutgers.eduDOI 10.15252/emmm.201404749

## Abstract

Increasing evidence suggests that the heart controls the metabolism of peripheral organs. Olson and colleagues previously demonstrated that miR-208a controls systemic energy homeostasis through the regulation of MED13 in cardiomyocytes (Grueter *et al*, 2012). In their follow-up study in this issue of *EMBO Molecular Medicine*, white adipose tissue (WAT) and liver are identified as the physiological targets of cardiac MED13 signaling, most likely through cardiac-derived circulating factors, which boost energy consumption by upregulating metabolic gene expression and increasing mitochondrial numbers (Baskin *et al*, 2014). In turn, increased energy expenditure in WAT and the liver confers leanness. These findings strengthen the evidence of metabolic crosstalk between the heart and peripheral tissues through cardiokines and also set the stage for the development of novel treatments for metabolic syndrome.

See also: **KK Baskin *et al*** (December 2014)

The functions of multiple organs located far apart can be regulated in a coordinated manner through neurohormonal communication. Recently, however, this classic endocrine mechanism has been dramatically expanded since novel factors secreted from organs previously assumed to be primarily non-endocrine, such as adipose tissues and skeletal muscle, have been shown to regulate the function of distal organs. The heart requires a considerable supply of energy for continuous pumping and continuously adapts to hemodynamic stress; it is therefore conceivable that heart-driven metabolic networks with peripheral organs are in place to achieve efficient coordination. For example, if the pumping function is reduced, the heart may signal peripheral organs to reduce oxygen and nutrient consumption. Alternatively, the heart may instruct peripheral organs to release energy substrates, such as fatty acids, to be delivered to the heart, thereby improving cardiac contractility.

Indeed, increasing evidence suggests that the heart is an organ that secretes proteins referred to as cardiokines, for inter-organ and inter-cellular communication. More than 16 secretory proteins have been identified thus to be cardiokines, including atrial natriuretic factor (ANF), B-type natriuretic peptides (BNP), angiotensin II, growth differentiation factor (GDF)-15, follistatin-like (Fstl) 1, myostatin, activin A, and Fstl3 (Shimano *et al*, 2012). These cardiokines play physiological and pathological roles in the regulation of growth, death, fibrosis, hypertrophy and remodeling. However, much less is known about the role of cardiokines in mediating metabolic crosstalk between the heart and peripheral tissues.

Natriuretic peptides are the most well-studied cardiokines and mediate natriuresis, diuresis, and vasodilation in the failing heart (de Bold, 2011). ANF also inhibits glycolysis, increases gluconeogenesis in the rat liver (Rashed *et al*, 1992), and regulates lipolysis and lipid mobilization in human adipocytes (Sengenes *et al*, 2000). Cardiac natriuretic peptides also upregulate PPARγ coactivator-1α (PGC-1α) and uncoupling protein 1 (UCP1) in adipocytes, leading to increases in mitochondrial biogenesis, thermogenesis, and energy expenditure (Bordicchia *et al*, 2012). Although the functional significance of the interaction between the heart and peripheral tissues through natriuretic peptides remains poorly understood, these observations suggest that the heart can regulate metabolism in the adipose tissue through cardiokines.

Recently, Eric Olson's group reported that the heart controls systemic energy metabolism, fat mass, and body weight via microRNA-208a (miR-208a) and Mediator complex subunit 13 (MED13) signaling (Grueter *et al*, 2012). miR-208a is encoded by an intron of the α*-myosin heavy-chain (MHC)* gene and is required for upregulation of β*MHC* and cardiac growth in response to pressure overload or hypothyroidism (van Rooij *et al*, 2007). MED13 is a direct target of miR-208a and as such negatively regulated by miR-208a. MED is a key component of the transcriptional machinery (Malik & Roeder, 2010). MED13 is one of about 30 mammalian MED subunits and comprises a kinase submodule with MED12, cyclin c, and cyclin-dependent kinase 8. MED13 controls gene transcription through thyroid hormone (TH) receptors and other nuclear hormone receptors that are known to regulate cardiac and systemic energy homeostasis (Huss & Kelly, 2004). Olson and colleagues found that in mice, inhibition of miR-208a or upregulation of MED13 in the heart confers leanness and resistance to diet-induced obesity through an increase in whole-body energy consumption. Conversely, genetic deletion of MED13 in the heart resulted in increased susceptibility to obesity. Whereas these findings clearly suggest that the heart signals to other tissues to alter their energy metabolic function, important questions remained: (i) What is the molecular mechanism by which cardiac MED13 regulates systemic energy metabolism? Is it mediated by cardiokines? (ii) If so, which organs are targeted by cardiokines to confer leanness? Olson and colleagues address these questions in their paper featured in this issue of *EMBO Molecular Medicine* (Baskin *et al*, 2014).

**Figure 1:**
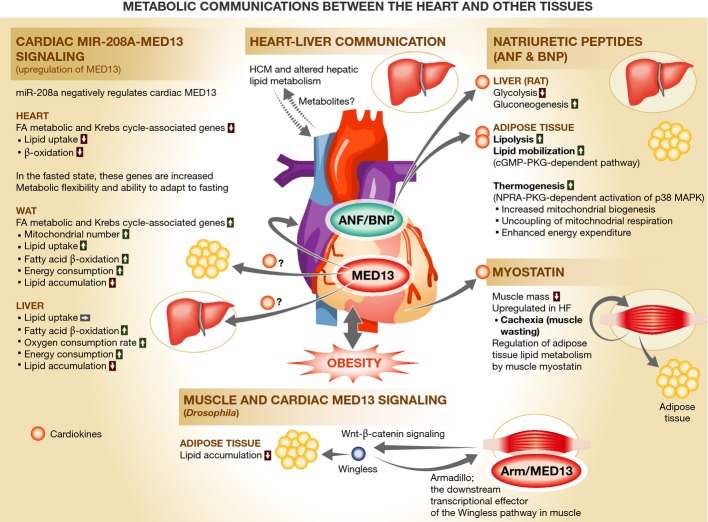
Metabolic communications between the heart and other tissues More than 16 proteins secreted from the heart have thus far been identified as cardiokines. Some of these act on other tissues to regulate metabolism in an endocrine manner. (A) Upregulation of MED13 in the heart enhances lipid utilization in white adipose tissue (WAT) and liver via unidentified circulating factors but decreases lipid utilization in the heart, thereby increasing systemic energy expenditure and leading to leanness (Baskin *et al*, 2014). (B) Metabolic derangement in a primary genetic heart disease, such as familial hypertrophic cardiomyopathy (HCM), adversely impacts liver metabolism (Magida & Leinwand, 2014). (C) Natriuretic peptides enhance lipolysis, lipid mobilization, and thermogenesis in adipocytes, in addition to natriuresis, diuresis, and vasodilation (Rashed *et al*, 1992; Sengenes *et al*, 2000; Bordicchia *et al*, 2012). (D) Secretion of myostatin (and probably other cardiokines, such as activin A) from the heart is increased in heart failure (HF) and accounts for muscle wasting (Shimano *et al*, 2012). Myostatin secreted from the skeletal muscle regulates lipid metabolism in adipose tissue (Lee, 2004). (E) Upregulation of MED13 in the heart and muscle increases the secretion of Wingless (Wnt), which enhances lipid metabolism in adipocytes in *Drosophila* (Lee *et al*, 2014). miR-208a, microRNA-208a; MED13, Mediator complex subunit 13; ANF, atrial natriuretic factor; BNP, B-type natriuretic peptide; NPRA, natriuretic peptide receptor A; PKG, cGMP-dependent protein kinase.

In cardiac-specific MED13-overexpressing mice (MED13cTg mice), MED13 increases systemic clearance of lipid from the blood by 60%. Despite the fact that MED13 was overexpressed only in the heart, lipid uptake, β-oxidation, mitochondrial content, and many genes involved in fatty acid metabolism and the Krebs cycle were increased in white adipose tissue (WAT) and liver. Although skeletal muscle accounts for approximately 30% of the resting whole-body metabolism (Zurlo *et al*, 1990), this was unaffected in MED13cTg mice. It is thus likely that WAT and liver are the targets of cardiac MED13 signaling and that induction of leanness is in part due to increased lipid utilization and oxygen consumption in the two organs. Furthermore, using heterochronic parabiosis, the authors show that the lean phenotype and the increased energy expenditure and mitochondrial activity in WAT and the liver were also induced in non-transgenic mice sharing their circulation with MED13cTg mice. These findings suggest that circulating factors secreted from the MED13cTg heart, most likely cardiokines, regulate metabolic gene expression and metabolic rates in WAT and liver, thereby leading to leanness.

These findings are novel and highly significant but two important questions await answers. First, what is the identity of the key circulating blood factors or cardiokines, and what are the mechanisms by which lipid metabolism is upregulated in WAT and liver? In the current paper, hematic levels of known metabolic hormones (such as insulin, adiponectine, thyroxine, and corticosterone), catecholamines, and natriuretic peptides are reported not to be altered. Recent evidence suggests that metabolic derangement in the heart caused by familial hypertrophic cardiomyopathy adversely impacts liver metabolism in part due to reduced lipid clearance from the blood by the heart. The consequent hepatic dysfunction in turn aggravates cardiac dysfunction (Magida & Leinwand, 2014). These results suggest that an inter-organ feedback mechanism may exist between the heart and the liver, probably through metabolites acting as soluble messengers, thus adversely affecting the each other's functions. In contrast, the current work demonstrates a favorable and positive regulation of WAT and liver fat metabolism by cardiac MED13 signaling, suggesting the involvement of cardiokines distinct from those involved in the negative regulation of the liver. The Olson group has recently identified Wingless, secreted from muscle via MED13 regulation, and Armadillo, the downstream transcriptional effector of the Wingless pathway, as key factors of adiposity in *Drosophila* (Lee *et al*, 2014). Wingless (Wnt) appears to be a soluble mediator of muscle MED13 signaling, and it decreases lipid accumulation in adipocytes. Interestingly, activation of the canonical Wnt-β-catenin pathway in adipose tissue was recently shown to decrease fat mass in mammals (Zeve *et al*, 2012). In aggregate, these findings suggest an evolutionarily conserved metabolic crosstalk between the muscle and adipose tissue. Whether or not the Wnt-β-catenin pathway mediates the effect of cardiac MED13 on the lean phenotype in mice remains to be elucidated.

Second, what are the physiological and pathological roles of endogenous MED13? Might endogenous cardiac MED13 signaling be regulated in response to metabolic stress, such as obesity and insulin resistance? The expression of miR-208a increases developmentally, in parallel with the switch in expression from the β-MHC to the α-MHC gene, coincident with a surge of circulating thyroid hormone shortly after birth (Callis *et al*, 2009). Since left ventricular heart failure is often accompanied by upregulation of β-MHC and therapeutic inhibition of miR-208a improves left ventricular cardiac function in Dahl hypertensive rats (Montgomery *et al*, 2011), one can speculate that miR-208a is upregulated and, thus, MED13 might be downregulated during heart failure. On the other hand, miR-208 is progressively downregulated in the right ventricle (RV), which, in turn, activates the MED13-NCoR1 pathway, inhibits myocyte enhancer factor 2, and exacerbates RV failure (Paulin *et al*, 2014). While the change in cardiac MED13 appears sufficient to induce metabolic effects in WAT and the liver, how it affects both cardiac and systemic metabolism during heart failure, the hallmarks of which are the heart running out of fuel and the presence of cachexia, remains to be elucidated (Neubauer, 2007). It should be noted that genetic deletion of miR-208a increases myostatin (known to be a cardiokine) in the heart (Callis *et al*, 2009; Shimano *et al*, 2012), which in turn induces cachexia, characterized by body and muscle wasting (Anker *et al*, 1997; Lee, 2004; Heineke *et al*, 2010), and increases mortality in patients with heart failure (George *et al*, 2010). In the current study, the authors report that upregulation of MED13 downregulates genes involved in β-oxidation and the TCA cycle. It would be interesting to establish whether the effect of MED13 on cardiac metabolism impacts on the function of cardiomyocytes and why MED13 differentially affects the cardiac muscle and peripheral organs.

Pharmacological interventions to modulate cardiac miR-208a-MED13 signaling or MED13-regulated cardiokines may provide therapeutically useful avenues in obesity, diabetes, dyslipidemia, and the other systemic metabolic disorders. Indeed, inhibition of miR-208a with LNA-anti-miR-208a conferred resistance to diet-induced obesity and glucose intolerance (Grueter *et al*, 2012). It should be noted, however, that genetic deletion of miR-208a decreased connexin 40 expression and induced arrhythmia, such as atrial fibrillation (Callis *et al*, 2009). Thus, it is necessary to carefully evaluate whether pharmacological interventions would similarly affect the cardiac conduction system and thus induce arrhythmia. The level of MED13 and cardiokines may be regulated by other parallel mechanisms in addition to miR-208a, which may therefore be considered should the modulation of miR-208a prove problematic and/or unfeasible.

In summary, Olson and colleagues identify WAT and liver as the target organs of cardiac MED13 signaling, which enhances energy consumption by increasing lipid metabolic gene expression and mitochondrial numbers. Parabiosis experiments suggest the existence of circulating blood factors, perhaps cardiokines, regulated by the miR-208/MED13 pathway. These findings do not only strengthen the evidence of metabolic crosstalk between the heart and peripheral tissues, but also bear therapeutic implications for systemic metabolic disorders, such as obesity.
